# Effects of Unfiltered Cigarettes on Smoking Behavior and Toxicant Exposure: Protocol for a Randomized Crossover Clinical Trial

**DOI:** 10.2196/19603

**Published:** 2020-12-08

**Authors:** Eyal Oren, Kim Pulvers, Devan R Romero, Casey Barber, Erika Carter, Laree A Tracy, Thomas E Novotny

**Affiliations:** 1 Division of Epidemiology and Biostatistics School of Public Health San Diego State University San Diego, CA United States; 2 Department of Psychology California State University San Marcos San Marcos, CA United States; 3 Department of Kinesiology California State University San Marcos San Marcos, CA United States

**Keywords:** nicotine, tobacco, topography, exposure, policy, environmental

## Abstract

**Background:**

Plastic filters on cigarette butts are a widespread source of nonbiodegradable, toxic environmental waste. State and local legislation to ban the sale of single-use cigarettes may be considered to prevent this waste, but scientific evidence on the impact of switching smokers to unfiltered cigarettes on smoking behavior and toxicant exposures is needed to inform this policy. We have designed an open-label, randomized, 9-week, crossover clinical trial of adult filtered-cigarette smokers who switch to unfiltered cigarettes.

**Objective:**

Our objective is to understand the impact of switching smokers of filtered cigarettes to unfiltered cigarettes on smoking behavior and toxic exposures.

**Methods:**

This trial involves a 1-week baseline period; a 2-week period of smoking filtered or unfiltered cigarettes, where groups are randomly assigned; a 3-week washout period; another 1-week baseline period; and a 2-week crossover period of smoking the opposite condition (ie, filtered or unfiltered cigarettes) for a sufficient sample size of 40 participants. We will determine changes in (1) observed topography (ie, puff count, interpuff interval, and puff volume) and cigarettes smoked per day, via butt counts and self-report, (2) expired carbon monoxide and excretion of urinary cotinine, 4-(methylnitrosamino)-1-(3-pyridyl)-1-butanol, and volatile organic compounds, and (3) participants’ knowledge and attitudes toward unfiltered cigarettes, satisfaction with smoking, and intention to quit if they were not able to smoke filtered cigarettes.

**Results:**

This study was funded in June 2018 and approved by the relevant Institutional Review Boards in July 2018. This study has enrolled 37 participants as of October 2020. Data analysis is currently underway, and trial results are expected to be published in spring 2021.

**Conclusions:**

This pilot proof-of-principle study will inform the design of a larger, future research project that can provide robust scientific evidence on our research question. Such a large study could inform possible state or local legislation to ban the sale of single-use filtered cigarettes in order to mitigate the environmental impact of discarded single-use plastic filters.

**Trial Registration:**

ClinicalTrials.gov NCT03749876; https://clinicaltrials.gov/ct2/show/NCT03749876

**International Registered Report Identifier (IRRID):**

DERR1-10.2196/19603

## Introduction

### Background and Rationale

Cellulose acetate (ie, plastic) filters on cigarette butts are a widespread source of nonbiodegradable environmental waste that may be toxic to marine organisms, aquatic environments, and possibly to human and animal health [1-4]. Cigarette butts are, in fact, a major littered item found during beach and urban cleanups each year throughout the world and adversely affect storm water drainage, beaches, neighborhoods, and other natural environments [5]. There is a widespread perception among smokers and nonsmokers that filters provide a *safer* cigarette [6]. In response to the expanding evidence in the 1950s regarding risks for lung cancer and other serious illnesses due to smoking, the tobacco industry sought to address or at least obfuscate these risks through the development of cigarette *filters*. This terminology suggested purification or reduction of smoking risks to the consumer [6]. It is clear that consumers responded to the marketing blitz around filters and the perceived risks of smoking such that today more that 99% of commercially sold cigarettes in the United States are filtered [7].

Most smokers and nonsmokers do not know that the filters attached to almost all cigarettes sold in the United States are made of nonbiodegradable plastic. In addition, cigarette filters have also been deemed “unproved in reducing harms to the average smoker” by the US Surgeon General and the National Cancer Institute [7,8]. Data comparing the relative risks of smoking among age-matched cohorts of smokers across 50 years show that overall mortality, as well as the smoking-attributable risks for morbidity and mortality from lung cancer, heart disease, and chronic obstructive pulmonary disease, increased over the years during which filters became standard issue as part of manufactured cigarettes [9,10]. State and local governments have jurisdiction to ban the sale of various tobacco products, and in 2019-2020, the California Assembly considered, for environmental reasons, a bill to ban the sale of single-use filtered cigarettes [11]. Additional scientific evidence on the human consequences of removing single-use filters from cigarettes is needed to understand what, if any, health and behavioral impacts may result from a sales restriction to eliminate cellulose acetate–filtered cigarettes from the tobacco market.

### Study Objectives

This is a pilot study that will inform a possible larger clinical trial. The research question for the overall project is as follows: *What is the impact of switching smokers of filtered cigarettes to unfiltered cigarettes on smoking behavior and toxic exposures?* The specific aims are as follows:

1. Determine smokers’ satisfaction and attitudes toward smoking cigarettes if they were to switch from smoking filtered to unfiltered cigarettes.
2. Measure changes in smoking topography (ST) (ie, puff count and puff volume) and cigarettes smoked per day, via butt counts and self-report, among smokers who change to unfiltered cigarettes for 2 weeks compared with these measures while smoking filtered cigarettes.
3. Measure changes in urinary cotinine, 4-(methylnitrosamino)-1-(3-pyridyl)-1-butanol (NNAL), and volatile organic compound (VOC) excretion among smokers who smoke unfiltered cigarettes for 2 weeks compared to smoking filtered cigarettes.

Study results may inform proposed state or local legislation to ban the sale of single-use filtered cigarettes in order to eliminate the environmental impact of improperly discarded, poorly degradable plastic cigarette filters as toxic waste.

### Overview and Trial Design

While the risks of smoking any type of tobacco product are now clear to the public, there has never been a clinical trial comparing changes in biochemically measured exposures, perceptions, topography, and other behavioral elements of cigarette smoking when switching from filtered to unfiltered cigarettes (ie, there have been no studies reported in PubMed or ClinicalTrials.gov as of October 2020). If large-scale trials were needed to definitively answer concerns that policy makers, smokers, and the general public have regarding any potential adverse effects of eliminating filtered cigarettes from the market, it would be important to ascertain the practicality and validity of measures used for such trials. General perceptions of filtered cigarettes and their safety, palatability, and composition have been measured through national panel surveys (publication in progress), but actual changes among smokers switching between filtered and unfiltered cigarettes can only be measured in a clinical trial. Further, to eliminate exposure bias, a trial in which the order of exposure is randomized is most appropriate.

We have completed an open-label, randomized, 9-week, two-sequence, two–treatment condition, crossover clinical trial of 37 adult filtered-cigarette smokers who switch to unfiltered cigarettes. We will evaluate this pilot study’s approach as to its applicability for a follow-up research project with a larger sample size.

This approach uses a crossover design, which allows participants to be their own matched control, hence removing participant-level variability [12]. Cigarette smoking is the *chronic* condition in this trial that persists throughout the washout periods, with changes measured against the chronic condition, not against a nonsmoking condition. This design is often used in clinical trials particularly when evaluating interventions to treat or control chronic diseases such as asthma, for which there is large variability across measures within participants. In this pilot trial, we are measuring changes in smoking behavior due to changes in the product smoked, while not assuming any change in the underlying condition of smoking; this study design then accounts for participant-specific variability. Participants were instructed to resume pretreatment activity (ie, smoking filtered Camel or Pall Mall cigarettes) during the washout period during which there were no study measurements. We assumed 3 weeks to be sufficient to *wash out* the effect of exposure to the study cigarettes during the active treatment period [13].

We collected behavioral data via validated computer-based surveys at baseline, intervention, and postintervention time points to assess changes in knowledge and attitudes regarding smoking of filtered and unfiltered cigarettes. We used a handheld smoking device, the Clinical Research Support System (CReSS) Pocket (Borgwaldt KC), to measure ST over five, daily, 8-hour periods per week; participants took home the device, and measurements were recorded in a naturalistic setting. The rationale for five, 8-hour, ST monitoring periods was based on our aim to measure ST during a representative period for naturalistic smoking. Findings from previous studies using ST measures have not differed between direct and indirect observations [14-16]. Although slightly greater ST variability has been reported between individuals smoking with a CReSS device compared to normal smoking, many studies report consistent within-participant comparisons [14,16,17]. Thus, we were not concerned that smoking outside of the monitoring window would differ from measurement periods. This device enables convenient recording of ST throughout the day and over weeks with time- and date-stamped data; it thus allows evaluation of changes in frequency and characteristics of smoking patterns under different treatment conditions. Participants collected and returned cigarette butts each week, and they self-reported the number of cigarettes smoked per day [18]. We measured exhaled carbon monoxide (CO) weekly, and we collected urine samples at baseline, before and after the washout period, and at the end of the study to measure creatinine-normalized cotinine, NNAL, and VOC excretion.

## Methods

Our study protocol followed the SPIRIT (Standard Protocol Items: Recommendations for Interventional Trials) guidelines (see Multimedia Appendix 1 for the SPIRIT checklist).

### Study Setting

The study was conducted in San Diego, California, United States; participants were screened remotely by Institution A and attended the smoking laboratory facility at Institution B in person. All biologic data and other measurements were collected and analyzed at either Institution A or B.

### Eligibility Criteria

#### Inclusion Criteria

Participants met all of the following criteria: aged 21-65 years; smokers of 5 or more cigarettes per day (CPD) on 25 or more days per month for 1 year or more; smokers of Camel or Pall Mall filtered cigarettes for at least two weeks prior to enrollment, and willing to continue using this brand during the study; fluent in English; have regular telephone access; and have transportation to attend all scheduled visits. Participants must have primarily (ie, ≥50% of the time) smoked cigarettes, allowing for secondary use of other tobacco products. We verified their regular smoking status upon enrollment with an expired CO level of at least 10 ppm [19]. Because we expected to be able to detect changes in our primary outcomes at lighter levels of smoking [20,21], we included light smokers in the study.

#### Exclusion Criteria

Participants were excluded if they were currently in a smoking cessation program or participating in another clinical trial; were using nicotine replacement therapy or medication that aids smoking cessation in the past month, including Zyban (bupropion), Catapres (clonidine), Pamelor (nortriptyline), or Chantix (varenicline); or were trying to quit or reduce smoking patterns in the past month. Women who were pregnant, breastfeeding, or planning to become pregnant in the next six months were excluded. Medical exclusion criteria included any of the following:

1. Self-reported uncontrolled diabetes mellitus or presence of any cardiovascular issue in the past 30 days, including heart attack, stroke, severe angina (ie, chest pain), hypertension, ischemic heart disease, vascular disease, or any other cardiovascular disease.
2. Presentation to the enrollment visit with a systolic blood pressure greater than 160 mm Hg or a diastolic blood pressure greater than 105 mm Hg, as verified by two consecutive blood pressure readings.
3. Self-reported hospitalization for psychiatric issues.
4. Being mentally or physically unfit to participate in the study.
5. Current dependence on a substance other than nicotine.

### Informed Consent and Ethics Approval

Informed consent was ensured during the smoking laboratory visit by trained research assistants. All consent statements were recorded in person. Participants were informed during the consent process that they may withdraw from the study at any time for any reason. Participants were also provided with information on the purpose of the study, study objectives, and how study success will be measured. The study was approved by the Human Research Protection Program from San Diego State University (approval number HS-2018-0152).

### Intervention Description

The first week involved baseline measurements of smoking behavior and urinary biologic markers. The next 2 weeks (Weeks 2 and 3) involved filtered- or unfiltered-cigarette smoking treatments, followed by a 3-week washout period (Weeks 4-6). Week 7 involved a postwashout, repeat baseline period. The crossover condition was implemented in Weeks 8 and 9. Study cigarettes were provided during the two treatment periods (ie, 4 weeks total). ST was measured on 5 days of smoking over 8-hour periods per day during the two baseline weeks (ie, enrollment and postwashout) and during the 4 weeks of switching trials; expired CO, weight, and survey measures were assessed at all visits.

### Study Cigarettes

Two brands of cigarettes are currently available as filtered and unfiltered: Camel and Pall Mall. Participants were provided supplies of one of these two brands throughout the study period based on their preference. After baseline measurements at the beginning of Week 2, we randomly assigned participants to smoke 2 weeks’ worth of filtered or 2 weeks’ worth of unfiltered study cigarettes. We supplied study cigarettes according to the average cigarettes smoked per day for the previous week plus 10%. For example, a participant who reported smoking 10 CPD in the previous week would be provided with a supply of cigarettes that would allow 11 CPD for the entire 2-week trial. Although it is possible that changes in CPD might result from increased supplies, participants were encouraged to smoke as normally as possible and to return any unused study cigarettes in order for us to measure changes in CPD resulting from switching.

### Encouraging Adherence

To support participants’ adherence to the interventions, study staff reviewed relevant expectations in detail for participants at each laboratory visit. A reminder card with the week’s instructions and next laboratory appointment was provided as well as a troubleshooting guide for operation of the CReSS Pocket device. Incentive payments were provided for returning cigarette butts as well as for correct usage of the CReSS device.

### Outcome Measurements

#### Knowledge and Attitudes Toward Unfiltered Cigarettes

Participants were asked at enrollment and at the final visit to what extent they believed that the filter on their brand of cigarettes (1) protects them from health problems caused by smoking and (2) makes smoking more pleasurable. Response options included the following: not at all, a little, quite a bit, and a great deal. In addition, they were asked “If filtered cigarettes were no longer available, what, if anything, would you change about your smoking patterns?” Response options included the following: increase number of cigarettes I smoke, nothing, cut back, and try to quit smoking altogether [22]. Participants were also asked about the purpose of filters on cigarettes, with the following response options (more than one answer was accepted): making cigarettes safer to smoke, making it easier to begin smoking, making it more pleasurable to smoke, selling more cigarettes, making cigarettes cheaper, and other. Finally, participants were asked what filters are made of, with the following response options (more than one answer was accepted): cotton, food starch, asbestos, plastic or cellulose acetate, and other [23,24].

Questions at enrollment and the final visit also covered the following: possible environmental consequences of smoking (eg, whether discarded butts are a problem for the environment and what should be done to prevent these consequences), if they had previously smoked unfiltered cigarettes, and what they would do if filtered cigarettes were no longer available for purchase.

At each lab visit, participants smoked a cigarette and answered questions from the Cigarette Evaluation Scale [25]. These questions included the following: Was it satisfying? Did it taste good? Did it make you dizzy? Did it calm you down? Did it help you concentrate? Did it make you feel more awake? Did it reduce your hunger for food? Did it make you feel nauseous? Did it make you feel less irritable? Did you enjoy the sensations of the smoke in your throat and chest? and Did it immediately reduce your craving for cigarettes? Each item was rated on a numbered 7-point Likert scale, ranging from 1 (not at all) to 7 (extremely). Subscales included Smoking Satisfaction, Psychological Reward, Aversion, Enjoyment of Respiratory Tract Sensations, and Craving Reduction. Response order was the same for all scales.

#### Intention to Quit

Participants were asked at each visit “What best describes your intentions to stop smoking completely, not even a puff?” Response options included the following: never expect to quit, may quit in the future but not in the next six months, will quit in the next six months, and will quit in the next 30 days [13]. In addition, they were asked whether they have currently set a limit for how many cigarettes they smoke per day to decrease health risks from smoking [26].

#### Nicotine Dependence

The Fagerström Test for Nicotine Dependence [27] was administered at enrollment and at the final visit, and a single-item index of addiction to cigarettes (0-100) [28] was established at every visit. The Brief Wisconsin Inventory of Smoking Dependence Motives was also assessed at enrollment (ie, baseline) and at the final visit [29].

#### Cigarettes Per Day: Self-Report and Butt Count

Standard survey questions measured how many days in the past month (enrollment [ie, baseline] visit), past week (weekly visits), and past 2 months (final visit) participants have smoked, as well as how many CPD they have smoked on those days (all study visits). In addition, participants returned their cigarette butts in a sandwich-size Ziploc bag or glass jar each week to provide a validation of their self-report. While these butts were disposed of as toxic waste in approved containers, a small portion were retained for future analyses. Previous studies have included butt counts with reliability set at 75% of returns [30]. We excluded butt count data from participants who did not return 75% of butts and provided incentive payments for returning cigarette butts at this level.

#### Smoking Topography

Behavioral adaptations were measured by ST, including puff number per cigarette, average puff duration (seconds), average interpuff interval (seconds), average flow rate (mL/s), and average and total volume (mL) [14]. We used the portable CReSS device to measure topography over five, daily, 8-hour periods per week: at baseline (Week 1), during the initial switch (Weeks 2 and 3), at postwashout baseline (Week 7), and again after the crossover switch (Weeks 8 and 9). Participants were trained in the use of the device by the study team at the initial lab visit in a specialized facility designed with proper ventilation to accommodate indoor smoking research. Multiple days of measurement allowed for assessment of reliability and sensitivity of topography changes as a function of the filtered and unfiltered cigarette switch. Topographic measures by CReSS have been compared to direct observation via video recordings [15]. One limitation of the CReSS device is inconsistent methodology and guidelines in calibration settings and standard usage, as well as established acceptable ranges of ST variables (eg, peak flow rates, interpuff interval, etc). However, past studies have mainly used manufacturer guidelines with added modifications and/or adaptations [31], and these were recommended in a recent review [32]; there are no changes in recommendations for newer devices.

#### Expired Carbon Monoxide

Expired CO provides a measure of exposure to tobacco smoke and other air pollutants. An expired CO breath test was conducted at each visit with the coVita Micro+ Smokerlyzer device (Bedfont) to assess expired CO levels.

#### Cotinine

Cotinine is the main proximate metabolite of nicotine. Urinary cotinine was measured at each visit by liquid chromatography–mass spectrometry (LCMS) [33] and normalized for urinary creatinine. The correlation between urinary and plasma cotinine is improved by adjusting the urinary cotinine levels for urinary creatinine concentration, which takes into account the variations in urinary dilution between samples [34].

#### Tobacco-Specific Nitrosamines

Tobacco-specific nitrosamines are carcinogens found in tobacco and tobacco smoke. Excretion of the carcinogen biomarker NNAL, which will be normalized for urinary creatinine, will be measured by LCMS [23] in urine samples. In addition, carcinogenic VOCs, excreted as mercapturic acids, will be measured from urine samples. Both are useful biomarkers of changes in exposure to tobacco smoke [35,36]. These metabolites, along with expired CO, provide biomarkers of exposure, thus creating a battery that reflects risks for smoking-induced diseases [37] that may vary between filtered and unfiltered cigarette smoking.

### Safety Monitoring Questions

Respiratory effects were assessed at every visit by asking participants about shortness-of-breath episodes or awakening from sleep due to breathing difficulties during the past 2 weeks (yes/no). Nicotine toxicity was assessed at every visit by asking whether the following symptoms were experienced in the last 2 weeks: nausea and/or vomiting, nervous irritability beyond normal day-to-day stresses, tremors, rapid heart rate, nightmares, and chest pains (yes/no). Blood pressure was also monitored at every visit.

### Criteria for Discontinuing or Modifying Allocated Interventions

Serious adverse events will result in discontinuation of the intervention; these are defined as any of the following:

1. Events that have resulted in death.
2. Events that are life threatening.
3. Events that require inpatient hospitalization.
4. Events that result in persistent or significant disability or incapacity.
5. Any other adverse event that, based upon appropriate medical judgment, may jeopardize the subject’s health and may require medical or surgical intervention to prevent one of the other outcomes listed in this definition.

Study personnel will notify the principal investigator (PI) of any serious adverse events immediately after first awareness of the problem. The PI will immediately report serious adverse events to the Institutional Review Board (IRB) and the Data Safety and Monitoring Board (DSMB).

### Participant Timeline

**Figure 1:**
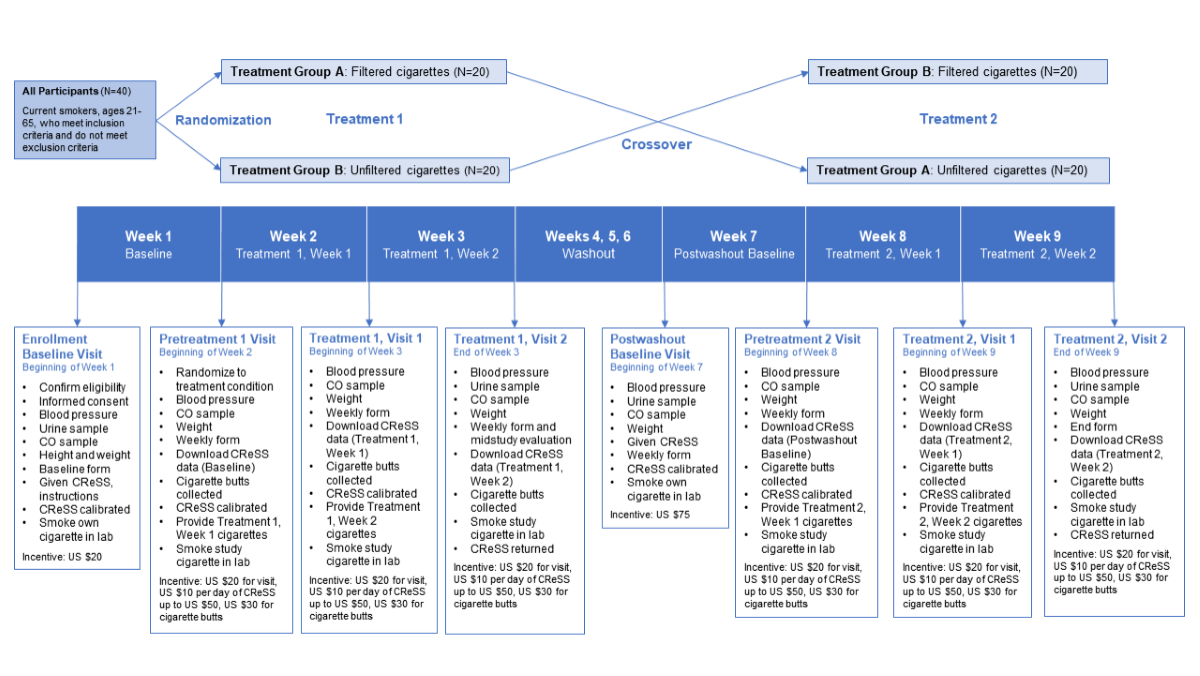
Study design diagram of the randomized crossover clinical trial of unfiltered cigarettes. CO: carbon monoxide; CReSS: Clinical Research Support System.

There will be a 1-week baseline period; a 2-week period of smoking filtered or unfiltered cigarettes, which will be determined at the time of randomization; and a 3-week washout period. This will be followed by a postwashout baseline week and a crossover to 2 weeks of smoking the opposite condition (see Figure 1).

### Sample Size

The study sample size is 40 participants, sufficient to detect moderate within-subject effect sizes (*f*=0.35) with a moderate correlation between repeated measures (*r*>0.50). This two-by-two crossover design has statistical power of 80% for a sample size of 20 in each group to detect an effect size of 10% change in average number of cigarettes smoked per day. We tried to recruit at least 10 participants of non-White ethnicities. If participants prematurely discontinued from the trial prior to crossover, we attempted to recruit replacements from the eligible pool or advertise for new participants. We recruited 20 women out of the 40 participants and randomized by sex. Sex stratification is of interest because men and women may metabolize smoke differently due to differences in body size and differences in cigarette preference and behavior. Any woman who becomes pregnant during the trial will be excluded from further participation [38].

### Recruitment

Recruitment was accomplished using a combination of Craigslist postings, newspaper ads, and paper flyers, which are approaches that have been successfully used in previous studies by the study team [39,40]. Prospective participants responded to advertisements via telephone or email to learn more about the study and to coordinate a time for phone screening to determine eligibility. Former participants in studies conducted at Institution B who have expressed interest in being contacted for future studies were contacted and invited to complete the screening questionnaire.

### Assignment of Interventions: Allocation

Once a participant was deemed eligible for the trial remotely by Institution A, trial personnel at Institution B followed up with the participant to schedule the initial trial appointment (ie, the enrollment, or baseline, visit). Participants who did not meet the eligibility criteria were not included in the trial. Recruitment and enrollment was a continuous process (ie, up to 10 participants at a time), which enabled us to utilize topography devices and lab facilities most efficiently. Randomization, stratified by sex, was based on a table of random numbers, and records were placed in sealed envelopes. A total of 20 participants were assigned to be first switchers following their enrollment (ie, baseline) visit at Institution B. These participants were then tested on individualized schedules (ie, not as a group), with each participant committing to 9 weeks of trial time, including a washout period at midtrial. All subjects had first smoked filtered Camel or Pall Mall cigarettes for at least two weeks prior to beginning the trial, according to the inclusion criteria. This standardized their exposure to a brand that was used as an unfiltered variety in the trial.

### Assignment of Interventions: Blinding

Investigators were masked to group allocation, but participants and research staff knew their exposure assignment due to the difficulties of blinding cigarettes that were filtered versus not filtered. DSMB members were unblinded to group allocation if needed.

### Plans for Assessment and Collection of Outcomes

There were eight scheduled lab visits, each with an incentive for attendance (see Participant Retention and Complete Follow-Up section). As shown in Table S1 in Multimedia Appendix 2, these visits were to occur weekly at baseline (Week 1), during the first 2 weeks of cigarette use (Weeks 2 and 3), during the postwashout baseline (Week 7), and during the second 2 weeks of cigarette use (Weeks 8 and 9). During each visit, participants were tested for expired CO, had blood pressure and weight measurements performed, and completed surveys (see Table S1 in Multimedia Appendix 2). At Weeks 1, 3, 7, and 9, participants provided a urine sample to measure normalized cotinine and carcinogen biomarkers. All collected urine samples were stored in a –80 °C freezer. Specimens were transported from Institution B to Institution A for laboratory testing.

During visits 2-4 and 6-8, participants visited the Institution B smoking laboratory facility with their topography device, cigarette butts, and unused cigarettes (left only on Weeks 3, 4, 7, and 8). Data from the topography device were downloaded so that the number of cigarettes logged in as smoked on the CReSS device could be compared to the total number of cigarettes or butts returned, as well as the number self-reported cigarettes smoked per day. Those deemed compliant with study procedures were paid for their time and supplied with another weeks’ supply of condition-assigned cigarettes. During visit 8, the same procedure was to be followed, except that it would be the final visit and no additional study cigarettes would be provided. Due to the COVID-19 pandemic, some of these procedures were curtailed and moved to remote follow-up.

### Participant Retention and Complete Follow-Up

Adherence to using the topography device was incentivized by paying participants US $10 per day for each day up to 5 days per week of use, checked at each lab visit. Compensation was provided for collecting and returning cigarette butts at US $30 per week for each week of participation. These payments, combined with payment for attending the laboratory visit (ie, US $20), totaled US $100 for all visits that were to occur during the 9 study weeks. For the postwashout baseline session, the compensation was US $75. The reimbursement schedule was reviewed with the participant at each visit (see Table S2 in Multimedia Appendix 2). In addition, weekly visit reminder cards were sent home with participants, which outlined next appointments and visit expectations (eg, collect all butts and use device for 5 days).

### Data Management

Study data were collected and managed using REDCap (Research Electronic Data Capture) tools that are hosted through Institution A. REDCap is a secure, web-based application that is designed to support data capture for research studies. All of the data collected were anonymized and password protected. Data were entered by trained study staff shortly after collection. All REDCap data are stored securely on a server at Institution A.

### Confidentiality

Confidentiality of data was assured by assigning code numbers to each participant survey. Participants’ identities are not linked to their responses. Any documentation of participant identities is kept in a locked filing cabinet located at the Institution A study office. Data are only accessible to personnel involved with this research, with access to servers limited by the research facility being locked at all times. All study information is maintained on secured computers and a password-protected laptop. The files on the laptop will be password protected for added security. The Institution A campus data network is protected by a perimeter firewall, and the network within the campus network is further protected by another institutional firewall.

### Statistical Methods

#### Primary and Secondary Outcomes

Descriptive statistics will be used to analyze baseline data to assess any imbalances between groups in baseline demographics and prerandomization characteristics. Stratified analyses and analyses of covariance will be performed to control for any baseline imbalances. To account for the crossover design and repeated measures, linear mixed-effects models with fixed effects for period and exposure sequence (ie, filtered then unfiltered or unfiltered then filtered) and with random effects for sex and participant will be used to analyze continuous outcomes and changes from baseline. Log transformation of nonnormal continuous measures will be performed when necessary. If data do not fit a linear model, generalized models will be considered. Model variance will be fit using a compound symmetry correlation structure with default degrees of freedom, which assumes constant variance between periods, with alternative covariance structures explored in the event of nonconvergence. For analysis of ordinal questionnaire data, we will assess for exposure and period effects using ordinal repeated-measures models with fixed effects for exposure sequence and period and with random effects for sex and participant. Prespecified cutoffs and thresholds for biomarkers and topography variables will be chosen based on a comprehensive literature review prior to conducting any analysis. Normalization and sensitivity analysis will be performed, if required. Changes in means for each continuous measure between participants and between weeks within treatment conditions will be calculated. Mean differences between exposure arms for these continuous measures will also be compared. *P* values and 95% confidence intervals will be reported when providing results from fitted models. All analyses will be performed using SAS 9.4 (SAS Institute) [41]. Noncomplier and dropout data will be utilized in mixed methods models up to the point of dropout and noncompliance for an intent-to-treat analysis. A completer-only (ie, per-protocol) analysis will also be run as a secondary analysis.

#### Missing Data

Missing data were to be minimized or avoided through extensive training of clinical research staff and repeated efforts to contact trial participants to obtain protocol-specified data. In the event of missing data, sensitivity analyses will be performed, including a completer analysis and multiple imputation approaches.

### Oversight and Monitoring

#### Safety Monitoring Plan and Adverse Event Reporting and Harms

This study was registered at ClinicalTrials.gov (NCT03749876). A DSMB charter was established outlining the board members’ responsibilities, meeting structure, deliverables, timeline, and membership requirements. A written schedule of their individual activities, phone numbers, and copies of their informed consent forms were provided to them. The DSMB members with relevant expertise who are not involved in this study included a physician, a clinical pharmacologist, and a health behavioral scientist appointed from Institution A or from other collaborating institutions. Data on serious adverse events, including severity, outcome, and management, were included in individual participant files and in aggregate form by treatment group and were reviewed weekly. These data were provided blinded to the DSMB in a detailed report by an Institution A research assistant. Potential problems reportable to the DSMB included self-reported health issues, such as respiratory problems, addiction or dependence, or nicotine toxicity signs and symptoms. In the event of concerns, the DSMB would notify the PI, and the medical consultant was to be promptly consulted. The PI would assess the potential risks to the participant regarding continuation of the trial and report back to the DSMB on findings; the DSMB would then decide if the trial should continue or if the participant should be excluded.

Although smokers will be switching to unfiltered cigarettes for this trial, a 2-week trial period is highly unlikely to provide any differential short- or long-term risks to participants who smoke unfiltered cigarettes. The targeted cigarette brands are commercially available to the population already and, hence, do not constitute a new product test. In addition, a physician-member of the research team will be on call to answer queries from the field staff regarding problems or questions from participants regarding switching to unfiltered cigarettes.

#### Auditing

All research staff received human subjects research training and appropriate training in recruitment, data collection, and management. Study progress was tracked with regular monitoring by the PI with study and clinic personnel, monthly research team meetings, and regular reviews with individual staff to ensure that study targets were being met.

#### Protections Against Risk

We measured biomarkers of carcinogen and nicotine exposures in order to assess the potential for long-term risk differences for unfiltered-cigarette versus filtered-cigarette smoking. In addition, because secondhand smoke is considered a risk exposure, and because this exposure is due to a combination of expired and side-stream smoke, this exposure to participants or their close contacts should be unaffected by the presence or absence of a filter.

We only recruited current smokers for this study, all of whom were also informed about the health consequences of smoking in the consent procedures. We trained study staff to be nonjudgmental toward smokers, to reassure them of anonymity in reporting findings, to reinforce the scientific value of this study, and to provide feedback after completion of the study. Both excluded individuals following the screening process as well as trial participants were referred for smoking cessation support to the state smoking cessation helpline. Any individuals deemed to have become ineligible, or those, for example, seeking to quit smoking, were provided with referrals and allowed to discontinue the trial.

#### Protocol Amendment Procedures

Any modifications or amendments to the study protocol were reviewed as a study team, discussed with the DSMB, and ultimately shared for approval by the IRB at Institution A prior to implementation.

### Dissemination Plans

This project is expected to result in a number of oral presentations of interest to local, state, and national stakeholders regarding possible regulatory actions on the sale of filtered cigarettes. In addition, results of this study will be published and presented at national and international meetings, such as those of the Society for Research on Nicotine and Tobacco, the American Public Health Association, the National Conference on Tobacco or Health, and joint scientific meetings of the Tobacco-Related Disease Research Program and California Department of Public Health.

### Availability of Data and Materials

The data sets used and/or analyzed during this study are available from the corresponding author on reasonable request.

## Results

This study was funded in June 2018 and was approved by the relevant IRBs in July 2018. This study has enrolled 37 participants as of October 2020. Data analysis is currently underway, and results are expected to be published in spring 2021.

## Discussion

This project will provide preliminary scientific evidence of the individual consequences of removing filtered cigarettes from the commercial tobacco market and how larger clinical trials of switching to unfiltered cigarettes may be undertaken. Outcomes and changes due to smoking unfiltered cigarettes include smokers’ satisfaction, attitudinal changes, changes in ST, changes in the number of cigarettes smoked per day, changes in exhaled CO, and changes in urinary cotinine and tobacco carcinogens. This is critical information that may be needed to inform and advance state or local legislation to ban the sales of filtered cigarettes. Such legislation would reduce the environmental impact of nonbiodegradable cigarette butt waste due to the cellulose acetate filter, although there might still be some environmental contamination from butt remnants. It is unknown whether eliminating filtered cigarettes from the tobacco product market will change smoking behavior, such that smokers will be more likely to quit or reduce cigarette consumption. Given that filters are essentially a marketing tool and not a health protective device, cigarette marketing success is likely to be reduced without this tool. If unfiltered cigarettes are less palatable than filtered cigarettes, it is likely that fewer cigarettes will be smoked and fewer children will become addicted. This study will provide missing information as to how smokers might react to no longer being able to smoke filtered cigarettes. If information about the lack of health protection of filters and their environmental impact becomes more widespread, fewer smokers may choose to smoke filtered cigarettes. The preliminary findings this study could be addressed in larger clinical trials in order to have a more substantial impact on tobacco product regulatory science more generally.

## Appendix

Multimedia Appendix 1 SPIRIT (Standard Protocol Items: Recommendations for Interventional Trials) checklist.

Multimedia Appendix 2 Study measures and time points, participant activities, and reimbursement schedule.

Multimedia Appendix 3 Peer-review report by the Tobacco-Related Disease Research Program (TRDRP).

## Abbreviations

CO: carbon monoxide
CPD: cigarettes per day
CReSS: Clinical Research Support System
DSMB: Data Safety and Monitoring Board
IRB: Institutional Review Board
LCMS: liquid chromatography–mass spectrometry
NNAL: 4-(methylnitrosamino)-1-(3-pyridyl)-1-butanol
PI: principal investigator
REDCap: Research Electronic Data Capture
SPIRIT: Standard Protocol Items: Recommendations for Interventional Trials
ST: smoking topography
VOC: volatile organic compound
