# Supplementary material for: Effects of Unfiltered Cigarettes on Smoking Behavior and Toxicant Exposure: Protocol for a Randomized Crossover Clinical Trial
Source: JMIR Res Protoc. 2020 Dec 8;9(12):e19603. doi: 10.2196/19603 (PMC7755531; doi:10.2196/19603)
Supplement: Multimedia Appendix 1 [file resprot_v9i12e19603_app1.docx]

**Administrative information: {x} corresponds to SPIRIT Guideline recommendation**

| Title {1} | Effect of Unfiltered Cigarettes on Smoking Behavior and Toxicant Exposure: Study Protocol for a Randomized Cross-over Clinical Trial |
| --- | --- |
| Trial registration {2a and 2b} | NCT03749876  https://clinicaltrials.gov/ct2/show/NCT03749876 |
| Protocol version {3} | January 2020, Version 1 |
| Funding {4} | This project is funded as a high-impact pilot grant through the University of California Tobacco-Related Disease Research Program. |
| Author details {5a} | Department of Psychology, California State University San Marcos  Department of Kinesiology, California State University San Marcos  School of Public Health, San Diego State University |
| Name and contact information for the trial sponsor {5b} | Tobacco-Related Disease Research Program (TRDRP)  Research Grants Program Office  University of California, Office of the President  300 Lakeside Drive, 6th Floor  Oakland, CA 94612  Phone: (510) 987-9870  Fax: (510) 587-6325  Url: www.trdrp.org |
| Role of sponsor {5c} | The study sponsor has no role in the design or implementation of the trial. |
