# Supplementary material for: Effects of Unfiltered Cigarettes on Smoking Behavior and Toxicant Exposure: Protocol for a Randomized Crossover Clinical Trial
Source: JMIR Res Protoc. 2020 Dec 8;9(12):e19603. doi: 10.2196/19603 (PMC7755531; doi:10.2196/19603)
Supplement: Multimedia Appendix 2 [file resprot_v9i12e19603_app2.docx]

## Multimedia Appendix 2: Tables

Table S1. Study Measures and Timepoints

|  | |  | | 1^st^ switch | |  |  |  | 2^nd^ switch | |
| --- | --- | --- | --- | --- | --- | --- | --- | --- | --- | --- |
| **List of Measures** | | Visit 1 | 2 | 3 | 4 | Washout | 5 | 6 | 7 | 8 |
| Questionnaires | |  |  |  |  |  |  |  |  |  |
|  | Demographics | x |  |  |  | *All smoke filtered*  *Pall Mall or Camels* |  |  |  |  |
|  | Smoking behavior, intention to quit | x | x | x | x |  | x | x | x | x |
|  | Knowledge and risk perceptions of filtered cigarettes | x | x | x | x |  | x | x | x | x |
|  | Fagerström Test for Nicotine Dependence | x | x | x | x |  | x | x | x | x |
|  | ATS Respiratory Symptoms | x | x | x | x |  | x | x | x | x |
|  | Modified Cigarette Evaluation Questionnaire | x | x | x | x |  | x | x | x | x |
|  | Brief Wisconsin Inventory of Smoking Dependence Motives | x |  |  |  |  |  |  |  | x |
| Height | | x |  |  |  |  |  |  |  |  |
| Weight | | x | x | x | x |  | x | x | x | x |
| Blood pressure | | x | x | x | x |  | x | x | x | x |
| Carbon monoxide | | x | x | x | x |  | x | x | x | x |
| Urinary cotinine and creatinine sample for analysis | | x |  |  | x |  | x |  |  | x |
| Urinary NNAL and VOC sample for analysis | | x |  |  | x |  | x |  |  | x |
| Smoking topography (measured on 5 days at home and downloaded at the following visit) | |  | x | x | x |  |  | x | x | x |
| Cigarette butt count (collected at home and returned at the following visit) | |  | x | x | x |  |  | x | x | x |

Table S2. Participant Activities and Reimbursement Schedule

| **Study Event** | **What Will Happen** | **Compensation** |
| --- | --- | --- |
| Smoke as usual for 2 weeks prior to study with Camel or Pall Mall filtered cigarettes | | |
| 1^st^ Lab Visit  Enrollment/Baseline  (1 hour)  **Beginning of Week 1** | - Confirm eligibility and enroll - Informed consent - Commitment to participate - Study measures - CReSS^a^ device calibration, training - Select days to use CReSS, schedule next visit - Smoke own cigarette in the lab using CReSS | **$20** |
| Smoke your own cigarettes for 1 week | Use CRESS on 5 days during the week  Collect cigarette butts^b^ |  |
| 2^nd^ Lab visit (1 hour)  Pre-treatment 1 Visit  **Beginning of Week 2** | - Study measures - Cigarette butts collected - CReSS data downloaded - Calibrate CReSS - Given first week of first type of study cigarettes - Select days to use CReSS, schedule next visit - Smoke study cigarette in the lab with CReSS | **Up to $100**  $10 per day of CReSS (up to $50) + up to $30 for cigarette butts + $20 for the lab visit |
| Smoke study cigarettes for 1 week | Use CRESS on 5 days during the week  Collect cigarette butts |  |
| 3^rd^ Lab visit (1 hour)  Treatment 1, Visit 1  **Beginning of Week 3** | - Study measures - Cigarette butts collected - CReSS data downloaded - Calibrate CReSS - Given second week of first type of study cigarettes - Select days to use CReSS, schedule next visit - Smoke study cigarette in the lab using CReSS | **Up to $100**  $10 per day of CReSS (up to $50) + up to $30 for cigarette butts + $20 for the lab visit |
| Smoke study cigarettes for 1 week | Use CRESS on 5 days during the week  Collect cigarette butts |  |
| 4^th^ Lab visit (1 hour)  Treatment 1, Visit 2  **End of Week 3** | - Study measures - Cigarette butts collected - CReSS data downloaded - Given instructions for washout - Return CReSS device - Schedule next visit | **Up to $100**  $10 per day of CReSS (up to $50) + up to $30 for cigarette butts + $20 for the lab visit |
| Washout: Return to pre-study smoking behavior for three-weeks (Camel or Pall Mall filtered)  **Weeks 4, 5, and 6** | | |
| 5^th^ Lab visit (1 hour)  Post-washout Baseline  **Beginning of Week 7** | - Study measures - CReSS given back to you and calibrated - Select days to use CReSS, schedule next visit - Smoke own cigarette in the lab using CReSS | **$75** |
| Smoke study cigarettes for 1 week | Use CRESS on 5 days during the week  Collect cigarette butts |  |
| 6^th^ Lab visit (1 hour)  Pre-treatment 2 Visit  **Beginning of Week 8** | - Study measures - Cigarette butts collected - CReSS data downloaded - Calibrate CReSS - Given first week of second type of study cigarettes - Select days to use CReSS, schedule next visit - Smoke study cigarette in the lab using CReSS | **Up to $100**  $10 per day of CReSS (up to $50) + up to $30 for cigarette butts + $20 for the lab visit |
| Smoke study cigarettes for 1 week | Use CRESS on 5 days during the week  Collect cigarette butts |  |
| 7^th^ Lab visit (1 hour)  Treatment 2, Visit 1  **Beginning of Week 9** | - Study measures - Cigarette butts collected - CReSS data downloaded - Calibrate CReSS - Given second week of second type of study cigarettes - Smoke study cigarette in the lab using CReSS | **Up to $100**  $10 per day of CReSS (up to $50) + up to $30 for cigarette butts + $20 for the lab visit |
| Smoke study cigarettes for 1 week | Use CReSS on 5 days during the week  Collect cigarettes butts |  |
| 8th Lab visit (1 hour)  Treatment 2, Visit 2  **End of Week 9** | - Study measures - Cigarette butts collected - CReSS data downloaded - Smoke study cigarette in the lab using CReSS - Return CReSS device - Tell us about your experience | **Up to $100**  $10 per day of CReSS (up to $50) + up to $30 for cigarette butts + $20 for the lab visit |

**^a^CReSS Pocket:** Handheld machine that you place your cigarette in while smoking; records “topography” such as puffing. We will send you home with the CReSS pocket so we can measure your smoking in your own environment as opposed to the lab environment. There are six weeks in which you will smoke using the CReSS Pocket, for an eight-hour period over five days. We will download your CReSS data at your lab visit and you will receive $10 per day that you do this, for up to $50 at a visit. We will determine whether the nicotine in your system matches the cigarettes per day that you report and your usage of the CReSS machine. Your CReSS incentive will be reduced if it appears that you are not using the machine as instructed. We will estimate a percentage of compliance and pay that amount. For example, if someone is 100% compliant they will receive full CReSS incentive. If someone is 50% compliant, they will receive 50% CRESS incentive. You are welcome to use the CReSS on more than five days and for longer than eight hours - we are requiring and incentivizing five days of eight-hour periods.

**^b^Cigarette Butt Count:** You will receive two separate two-week supplies of study cigarettes: one set will be filtered, and one set will be unfiltered. You will keep the butts from these cigarettes in a sandwich-sized Ziploc bag or glass jar to be brought to each of your weekly lab visits during the active four-week study period. You will receive $30 for bringing your used cigarette butts when they are due. Your butt incentive will be reduced if it appears that you are not collecting your butts as instructed.
