# Supplementary material for: Effects of Unfiltered Cigarettes on Smoking Behavior and Toxicant Exposure: Protocol for a Randomized Crossover Clinical Trial
Source: JMIR Res Protoc. 2020 Dec 8;9(12):e19603. doi: 10.2196/19603 (PMC7755531; doi:10.2196/19603)
Supplement: Multimedia Appendix 3 [file resprot_v9i12e19603_app3.pdf]

Applicant: Oren, Eyal
Title: A Randomized Crossover Clinical Trial of Unfiltered Cigarettes
Program: High Impact Pilot Research Award
Institution: San Diego State University Research Foundation
App #: 580582

View Review Information

Close Window Print

Committee: TRDRP 2018B APP - Social Behavioral, Treatment

Scoring range for this committee: 1 Exceptional (Best) - 9 Poor (Worst)

Summary Statement

Overall Score:

Reviewer Role: Primary Reviewer (457092)

Reviewer Summary: The proposed research study seeks to assess the acceptability among committed smokers of switching to unfiltered cigarettes from filtered cigarettes and then compare the measurement of exposure to nico ne and carcinogens after study par cipants make the switch. The study aims are to determine the attitudes towards smoking unfiltered or filtered cigarettes; to measure changes in smoking habits and cigarettes smoked per day among smokers who changed from unfiltered to filtered; and measure changes in urinary co nine and carcinogens in smokers after two weeks of switching from unfiltered to filtered cigarettes. The study proposes an open-label, randomized, 8 weeks, two-sequence, two-treatment, cross over clinical trial of 40 adult filtered cigarette smokers who switch to unfiltered cigarettes. The protocol for the study is to have a 1- week baseline period, 2-weeks of smoking filtered or unfiltered cigarettes, a 3-week washout period, and crossover to 2-weeks of smoking the opposite condi on.

Overall this was a well written proposal. The aims of the study were clearly presented. In additon, the study design was strong and the outcome of the research has great policy implica ons.

Criterion 1

PleasetiscoreandcommenttiOn:1)tiResponsivenesstoIntenttioftheAwardType(PilotProject);2)Innovation: Responsiveness to Intent of the Award Type (Pilot Project): The proposal is responsive to the intent of the award. The study proposed has implica ons for three of TRDRP's priori es including cancer preven on, environmental exposure and tobacco

control policy.

Criterion 2

Please score and comment on: 1) Research Plan; 2) Near-Term Leveraging Potential:

• The aims of the study are clear and presented well.
• During the washout period are smokers allowed to smoke? If not, what are the mechanisms in place to keep smokers from using tobacco products for three weeks?
• I'm not sure that collecting cigarette butts from participants will assure adherence to the study.
• The inclusion and exclusion criteria are adequate and appropriate for the study.
• The recruitment process is too reactive (i.e. flyers, newspapers, Craigslist, word of mouth). I think that a community partner (i.e. CBO) that could assist with recruitment would aid in getting study participants quickly.
• Incentives for the participants are adequate and may help with retention of study participants.
• How will the research team ensure that the study participants are only using the provided cigarettes? Smokeless tobacco, cigars, hookah and/or marijuana (blunts) can produce inaccurate levels of cotinine in which the study is only looking for levels from the products provided.

Near-term Leveraging Potential: If successful, the research from this trial could inform a larger clinical trial that can be submitted to the NIH/Food and Drug Administration and the data can serve as evidence for potentially banning the sale of filtered cigarettes.

Criterion 3

Please score and comment on: 1) Investigators; 2) Environment; 3) Community Engagement & Communication Plan:

Investigators: The PI has extensive experience in respiratory health and has primarily focused on respiratory infections, asthma, and chronic obstructive pulmonary disease, all of which are exacerbated by tobacco use. But he does not appear to have direct experience carrying out a study related to tobacco use, nicotine dependence, etc. However, the co-investigators have experience in tobacco epidemiology, environmental outcomes of smoking (i.e. butt waste) and nicotine dependence. The team in its entirety is a strength.

Environment: The environment seems adequate for the proposed study.

Community Engagement & Communication Plan: The study team may want to consider non-traditional ways of disseminating research results besides presenting at local and national events. The results can be shared via traditional and social media, blog posts and op-eds. In addition, the community engagement plan is heavily focused on cigarette butt pollution. Although cigarette butt pollution is an important factor in the study, the implications are far-reaching for policy, cancer prevention, and cessation. Community groups should be added that can provide input in those areas as well. A strength of the plan is utilizing NGO's such as the Truth Initiative (support letter included) and federal agencies such as CDC, NIH, and FDA to disseminate research findings.

Unscored Criterion

Comment on 1) Protection of Human Subjects from Research Risk, 2) Inclusion of Women, Minorities and Children in Research, 3) Care and Use of Vertebrate Animals in Research, 4) Biohazards, 5) Budget Concerns:

Protection of Human Subjects from Research Risk: No concerns in regard to the protection of study participants.

Inclusion of Women, Minorities and Children in Research: Inclusion of women are adequate. Representation of minorities are adequate considering the limitations of other ethnic groups being represented in the area. Children are not a part of the study.

Care and Use of Vertebrate Animals in Research: Not applicable

Biohazards: Not applicable

Budget Concerns: There are 14 people charged to the grant. It appears to be a lot of personnel for a study that only has 40 participants. In addition, there is not an adequate explanation as to why each of these positions are critical to carrying out the study. The research team may want to consider downsizing the number of co-investigators (5) and the number of grad students (4). In addition, is 3% of effort on the grant an adequate amount for the Project Manager to fulfill all of its duties and responsibilities to the research study?

Reviewer Role: Secondary Reviewer (76120)

Reviewer Summary: Well written proposal that is innovative and test a proof of concept using a rigorous cross-over design. By assessing smoker attitudes, topography and biomarkers, this proposal will provide novel information regarding switching to unfiltered cigarettes. The leveraging potential is high and study findings may have substantial policy implications. Research plan is well described and the research team has sufficient expertise and experience to accomplish the study objectives.

Criterion 1
Please score and comment on: 1) Responsiveness to Intent of the Award Type (Pilot Project); 2) Innovation:
Highly innovative pilot proposal that is very responsive to the pilot project award type and to TRDRP priorities. The proposal has potential to inform regulatory science and this line of work has substantial policy implications specifically regarding banning the sales of filtered cigarettes.

Criterion 2
Please score and comment on: 1) Research Plan; 2) Near-Term Leveraging Potential:
A rigorous cross-over design with washout period is proposed. The specific aims are well explicated and scientifically justified. Sample size justification is appropriate.

Overall, the study procedures are sufficiently described. However, the timeline is a bit confusing and figure 1 does not line up with table 1. Might be better to use days to illustrate.

The near-term leveraging potential is high as this work could provide preliminary data and inform a larger future trial of switching to unfiltered cigarettes.

Criterion 3
Please score and comment on: 1) Investigators; 2) Environment; 3) Community Engagement & Communication Plan:
Outstanding investigator team with strong and supportive institutional environment.
The community engagement and communication plan is excellent. Strong letters of support including from the Truth Initiative.

Unscored Criterion
Comment on 1) Protection of Human Subjects from Research Risk, 2) Inclusion of Women, Minorities and Children in Research, 3) Care and Use of Vertebrate Animals in Research, 4) Biohazards, 5) Budget Concerns:
No concerns noted

Reviewer Role: Tertiary Reviewer (297512)

Reviewer Summary: -An important study that will advance research addressing a number of TRDRP priorities.
-Study may likely impact Endgame policy decisions.
-Prior pilot testing and team work among investigators is strong.

Criterion 1

Please score and comment on: 1) Responsiveness to Intent of the Award Type (Pilot Project); 2) Innovation:

1) The proposed study is highly responsive to the intent of the pilot award. It addresses TRDRP priorities and the project may impact Cancer prevention, Treatment, and Biology; Environmental Exposure and Toxicology; and State and Local Tobacco Control Policy Research.

2) The study is significant since research has indicated increased risk of filtered cigarettes on adenocarcinoma of the lung. Additionally, the 2014 SGR indicated that there is suggestive evidence that ventilated filters increased levels of tobacco-specific nitrosamines, so more research is clearly needed in this area. Also, waste (butts) may be a toxic risk for humans, pets, and marine life – this is an environment problem. The study is innovative in that it uses an interesting assessment of smoking topography in the natural environment (Pocket CReSS) instead of lab study. Applicants discuss that results also may potentially lead directly to policy changes, including potentially banning filters from US cigarettes, similar to calls for menthol ban, and recent bans on light, mild advertising. Essentially, results could potentially eventually become directly incorporated into similar Tobacco Endgame policy strategies.

Criterion 2

Please score and comment on: 1) Research Plan; 2) Near-Term Leveraging Potential:

1) Research Design

Study Description:

A randomized cross-over pilot clinical trial of committed smokers switching from filtered cigarettes to unfiltered over two weeks, that will measure topography and attitudes of smoking these cigarettes.

Aim 1: Hypothesize smokers will have less satisfaction with unfiltered compared to filtered cigarettes.

Aim 2: Hypothesize they will inhale less deeply, frequently, and smoke fewer cigarettes/day.

Aim 3: Hypothesize they will have lower urinary cotinine, NNAL, and VOC excretion.

Strengths:

-Prior pilot testing of CReSS device for topographic measurement.

-Measures of CO testing are important to look at smoking overcompensation.

-Additional measures of biomarkers appear appropriate.

-Patient consent forms in Appendix appear appropriate.

Weaknesses:

-Don't appear to exclude cannabis smokers, which could impact results.

-Don't appear to measure for exposure to second-hand smoke, which could impact biomedical results.

2) Near-term leveraging potential

Applicant hopes to leverage results by informing a larger NIH/FDA trial that may potentially have policy impact. It is likely that knowledge gained through this proof of principle trial with inform the FDA, State and local officials and other tobacco researchers about the feasibility of conducting a larger-scale clinical trial of unfiltered cigarettes.

Criterion 3

Please score and comment on: 1) Investigators; 2) Environment; 3) Community Engagement & Communication Plan:

1) Investigators have collaborated on previous field test of the CReSS devices for the proposed research in the past with a Legacy Foundation grant. PI appears to have little experience in tobacco research directly, having mainly worked on pulmonary projects. However, applicant has assembled an excellent team of researchers including collaboration with Dr. Benowitz's group who are involved in the Data and Safety Monitoring Board. Dr. Novotny has been involved in Cigarette Butt Pollution project and SG reports. Strong letters of support from Dr. Samet (involvement also with SG reports) and Dr. Cummings will help with dissemination of research results.

2) Environment appears suitable, with the study taking place in Dr. Pulver's lab and samples results are sent to Dr. Benowitz's group.
3) Aspects of the local community engagement plan seem underdeveloped. For example, there is not a lot written about plans to engage the community in feedback about the project other than a discussion of how to recruit for the study. There is a plan to coordinate with policy folks who have recently introduced legislation to ban the sale of filtered cigarettes in California and collaborate with others on the Trash Amendment of the Clean Water Act. On the other hand, strength of the application includes plans to coordinate with FDA regulators, state agencies, Truth initiative, etc., which likely will assist with disseminating results on national scale.

Unscored Criterion

Comment on 1) Protection of Human Subjects from Research Risk, 2) Inclusion of Women, Minorities and Children in Research, 3) Care and Use of Vertebrate Animals in Research, 4) Biohazards, 5) Budget Concerns:

1) Human subjects seem protected
2) Includes women and minorities
